# The effect of wearing insoles with a toe‐grip bar on occupational leg swelling and lower limb muscle activity: A randomized cross‐over study

**DOI:** 10.1002/1348-9585.12193

**Published:** 2020-12-22

**Authors:** Hideki Nakano, Shin Murata, Yoshihiro Kai, Teppei Abiko, Dai Matsuo, Michio Kawaguchi

**Affiliations:** ^1^ Department of Physical Therapy Faculty of Health Sciences Kyoto Tachibana University Kyoto Japan; ^2^ ASICS Trading Company Limited Kobe Japan

**Keywords:** chronic venous disease, insole, lower limb muscle activity, occupational leg swelling, toe‐grip bar

## Abstract

**Objective:**

Sitting or standing for hours decreases the blood flow in the legs and results in increased pressure on the veins, leading to the development of chronic venous disease. This study aimed to investigate the effects of insoles with a toe‐grip bar on occupational leg swelling and lower limb muscle activity.

**Methods:**

This randomized cross‐over study enrolled 12 healthy men who work in a sitting or standing position. They were randomly divided into groups A (wore shoes with insoles with a toe‐grip bar for 8 hours each) and B (wore shoes with regular insoles for 8 hours each). After 1 week, groups A and B wore shoes with regular insoles and shoes with insoles with a toe‐grip bar, respectively, for 8 hours each. Lower leg volume was measured before and after each intervention, and lower limb muscle activity was measured at the start of each intervention.

**Results:**

Occupational leg swelling was significantly smaller in men wearing insoles with a toe‐grip bar (*P* < .05). Moreover, the integrated electromyogram value of the tibialis anterior muscle and medial and lateral gastrocnemius muscles during the stance phase of walking, and tibialis anterior muscle during the swing phase of walking was significantly greater in men wearing insoles with a toe‐grip bar (all *P* < .05).

**Conclusion:**

Insoles with a toe‐grip bar contribute to increased lower limb muscle activity, attenuating occupational leg swelling.

## 1 INTRODUCTION

It has been reported that occupational leg symptoms, such as leg swelling, leg pain, and the feeling of heaviness and tension in the legs, occur frequently in healthy individuals who work for hours at sitting and standing positions.^1^ Sitting or standing for hours decreases the blood flow in the legs and results in increased pressure on the veins; therefore, prolonged sitting and standing are both risk factors for the development of chronic venous disease.^2^ Occupational leg symptoms, especially leg swelling is associated with feelings of tiredness and heaviness of the legs, and leg pain.^1^, ^3^, ^4^ Therefore, reducing leg swelling is important in preventing the development of chronic venous disease.

Previous studies reported that compression therapy,^1^, ^4^, ^5^, ^6^, ^7^, ^8^, ^9^ electrical stimulation,^9^, ^10^ and leg movement^11^, ^12^ are effective in reducing occupational leg swelling. These methods promote skeletal muscle pump function, peak venous velocity, and venous return, thereby reducing ambulatory venous pressure, interstitial fluid volume, and leg volume.^13^, ^14^, ^15^ However, it has been reported that compression therapy is uncomfortable for the participants and is associated with side effects such as sweating, itching, and skin dryness.^16^, ^17^, ^18^ Similarly, electrical stimulation has been reported to cause adverse effects such as muscle tearing, tissue burn, and skin irritation.^19^ Moreover, leg movement requires regular and continuous exercise to be effective.^20^

To address these problems, insoles with a toe‐grip bar, which improve the toe‐grip strength and reduce leg swelling while the individual is walking, have been developed recently.^21^, ^22^, ^23^ According to some studies, the toe‐grip bar, which is a convex structure, is placed at the central part of the proximal phalanx from the first to the fifth toe.^21^, ^22^ During the terminal stance phase of walking, the toes perceive the toe‐grip bar and the reflex toe‐grip movement is increased; therefore, the toe‐grip movement occurs subconsciously when the individual walks. It has been reported that the insoles with a toe‐grip bar improved the toe‐grip strength in healthy individuals^21^, ^22^ and that it attenuates leg swelling in middle‐aged and elderly women.^23^ This may be due to the promotion of the skeletal muscle pump function by using the insoles. A previous study reported on the muscle activity of the rectus femoris, biceps femoris, tibialis anterior, and gastrocnemius muscles during the toe‐grip movement in a standing position.^24^ Therefore, we hypothesized that insoles with a toe‐grip bar may increase lower limb muscle activity, resulting in the attenuation of leg swelling.

This study aimed to investigate the effects of insoles with a toe‐grip bar on occupational leg swelling and lower limb muscle activity.

## 2 MATERIALS AND METHODS

### 2.1 Participants

Twelve healthy men (mean age ± standard deviation: 40.0 ± 8.8 years; mean height: 171.8 ± 2.4 cm; mean body weight: 66.9 ± 8.0 kg) who work in a sitting or standing position were included in this study. Participants with Mini‐Mental State Examination scores below 24 and orthopedic, neurological, cardiovascular, or psychiatric diseases that might influence the results were excluded. Only male participants were recruited because the toe‐grip strength and the muscle activity differed in terms of sex.^25^, ^26^, ^27^

The study was conducted according to the principles of the Declaration of Helsinki and was approved by the Institutional Ethics Committee of Kyoto Tachibana University. All participants provided their written informed consent, and they were allowed to withdraw from the study at any time.

### 2.2 Study protocol

This research was designed as a randomized cross‐over study. Initially, the participants were randomly divided into two groups (A and B) using random numbers generated by Microsoft Excel 2010 (Microsoft). Group A wore shoes with insoles with a toe‐grip bar, and group B wore shoes with regular insoles, for 8 hours each. After 1 week, group A wore shoes with regular insoles, and group B wore shoes with insoles with a toe‐grip bar, for 8 hours each. All participants were blinded to their group assignment.

**FIGURE 1:**
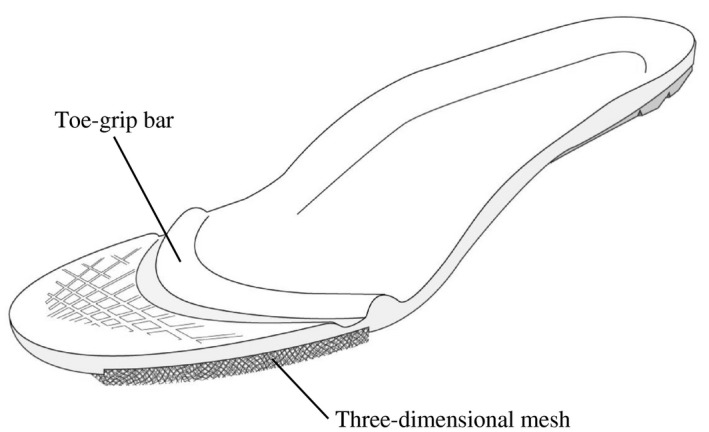
The insole design. The insoles used in this study consisted of a toe‐grip bar and a three‐dimensional mesh

**FIGURE 2:**
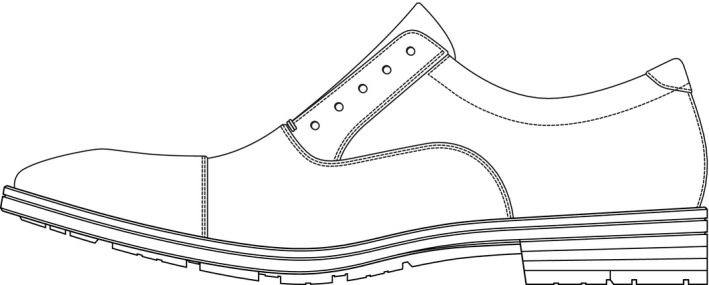
The business shoe design used in this study

The mid and rear parts of the insoles with a toe‐grip bar were made of synthetic resin foam, and the toe section was made from synthetic fiber with high‐repulsion properties (known as three‐dimensional mesh). The toe‐grip bar, which is a convex structure, was placed at the central part of the proximal phalanx from the first to the fifth toe (Figure 1).^21^, ^22^, ^23^ The regular insoles had no toe‐grip bar or a toe section made from synthetic fiber with high‐repulsion properties. The structure of the regular insoles was the same as that of the insoles with a toe‐grip bar. Except for the insoles, both groups wore the same standard business shoes provided by the study investigators (Figure 2). Each day, the study investigators provided the shoes to the participants in the morning and retrieved them 8 hours later.

### 2.3 Measurements

Lower leg volume was measured before and after each intervention, and the number of steps during each intervention was measured. Moreover, lower limb muscle activity was measured at the start of each intervention.

Lower leg volume was measured using the water displacement method.^28^, ^29^, ^30^ The reliability of the water replacement method has been reported in previous studies.^28^, ^29^ For the measurement, a rectangular‐shaped aquarium, with walls made of an acrylic board (length 300 mm × width 150 mm × height 450 mm, Natsume Seisakusho Co., Ltd.), was used. A drainage outlet was attached to the upper end of the aquarium, and any water overflow was discharged from the drainage outlet. Next, the participants gradually lowered their right lower limb into the full water tank, and the displaced overflowing water volume was measured using a measuring cylinder. The measurements were performed on the left and right lower limbs, and the average lower limb volume was calculated. The difference between the lower leg volume measured at the end of the intervention and the baseline lower leg volume in the morning was referred to as “occupational leg swelling”^5^, ^7^ and was used for the analysis.

The number of steps was measured using a pedometer with a 3‐axis accelerometer sensor (EX‐500; Yamasa Tokei Keiki Co., Ltd.).^31^ The pedometer was clipped to a belt at the waist and kept in place for 8 hours while the participants wore the insoles with a toe‐grip bar or regular insoles. The number of steps during each intervention was used for the analysis.

Lower limb muscle activity was measured using a wireless surface electromyography (EMG) system (Telemyo G2, Noraxon USA Inc).^32^ EMG was recorded from the right tibialis anterior muscle and the right medial and lateral gastrocnemius muscle based on the method described by SENIAM,^33^ at a sampling rate of 1000 Hz. A reference electrode was attached to the right head of the fibula. Initially, the maximum voluntary contraction (MVC) of each muscle was measured according to the manual strength test.^34^ Then, each muscle activity during walking was measured. A bandpass filter (20‐500 Hz) was employed to remove noise from the measured EMG data using an analysis software (MyoResearch XP, Noraxon USA Inc). After performing full‐wave rectification processing and time normalization with one walking cycle as 100%, the integrated electromyogram (IEMG) values of the stance and swing phases during walking were calculated. The calculated IEMG values were normalized based on the MVC of each muscle. The IEMG value of each muscle during the stance and swing phases when the participants walked wearing the shoes with a toe‐grip bar or the regular insoles was used for the analysis.

### 2.4 Statistical analyses

Baseline lower leg volume, occupational leg swelling, and the number of steps were analyzed using the Wilcoxon signed‐rank test. The IEMG values were analyzed using the paired t‐test. Statistical analyses were performed using SPSS 24.0 (IBM Corp.). The level of significance was set at <5%.

## 3 RESULTS

There were no reports of pain or discomfort due to the toe gripping bars, and all participants completed the cross‐over study. Occupational leg swelling was significantly smaller in men wearing insoles with a toe‐grip bar than in those wearing regular insoles (*P* < .05). There was no significant difference between insoles with a toe‐grip bar and regular insoles in terms of baseline lower leg volume and the number of steps (all *P* > .05) (Table 1). The IEMG value of the tibialis anterior muscle and medial and lateral gastrocnemius muscles during the stance phase of walking, and the tibialis anterior muscle during the swing phase of walking was significantly greater in men wearing insoles with a toe‐grip bar than in those wearing regular insoles (all *P* < .05) (Table 2).

**TABLE 1:** Comparison of the baseline lower leg volume, occupational leg swelling, and the number of steps between insoles with a toe‐grip bar and regular insoles

Parameters	Insoles with a toe‐grip bar	Regular insoles	*P*‐value
	Mean ± SD	Mean ± SD	
Baseline lower leg volume (mL)	3414.17 ± 304.76	3403.75 ± 293.98	.48
Occupational leg swelling (mL)	34.58 ± 41.69	85.42 ± 64.19	.02*
Number of steps (step)	3916.50 ± 1060.27	3661.25 ± 911.80	.35

Abbreviation: SD, standard deviation.

* *P* < .05, significant difference between insoles with a toe‐grip bar and regular insoles.

**TABLE 2:** Comparison of the lower limb muscle activity during walking between insoles with a toe‐grip bar and regular insoles

Parameters		Insoles with a toe‐grip bar	Regular insoles	*P*‐value
		Mean ± SD	Mean ± SD	
Stance phase	TA (IEMG)	21.71 ± 8.97	16.85 ± 7.56	.01*
	MG (IEMG)	28.34 ± 13.33	23.68 ± 9.54	.01*
	LG (IEMG)	28.74 ± 15.21	23.93 ± 13.17	.04*
Swing phase	TA (IEMG)	29.32 ± 9.46	22.20 ± 7.68	.00*
	MG (IEMG)	10.94 ± 6.32	12.54 ± 6.35	.13
	LG (IEMG)	8.79 ± 5.99	8.82 ± 5.36	.95

Abbreviations: IEMG, integrated electromyogram; LG, lateral gastrocnemius muscle; MG, medial gastrocnemius muscle; SD, standard deviation; TA, tibialis anterior muscle.

* *P* < .05, significant difference between insoles with a toe‐grip bar and regular insoles.

## 4 DISCUSSION

This study investigated the effects of insoles with a toe‐grip bar on occupational leg swelling and lower limb muscle activity. Based on the results, occupational leg swelling was found to be significantly smaller in men wearing insoles with a toe‐grip bar than in those wearing regular insoles. However, there was no significant difference in the baseline leg volume and the number of steps between two insoles. These results suggest that the insoles with a toe‐grip bar are effective in attenuating occupational leg swelling.

According to previous studies, compression therapy,^1^, ^4^, ^5^, ^6^, ^7^, ^8^, ^9^ electrical stimulation,^9^, ^10^ and leg movement^11^, ^12^ have been used to reduce occupational leg swelling. Compression therapy attenuates leg swelling by increasing tissue pressure,^1^, ^4^, ^5^, ^6^, ^7^, ^8^, ^9^ whereas electrical stimulation and leg movement attenuate leg swelling by reducing capillary pressure and increasing tissue pressure by increasing the skeletal muscle pump function.^5^, ^6^, ^11^, ^12^ In this study, the muscle activity of the tibialis anterior muscle and the medial and lateral gastrocnemius muscles during the stance phase of walking was significantly greater in participants wearing insoles with a toe‐grip bar. These results suggest that increasing the skeletal muscle pump function in the lower legs by using insoles with a toe‐grip bar attenuated occupational leg swelling.

In this study, the occupational leg swelling when using the insoles with a toe‐grip bar was 34.58 mL. According to a previous study that used the same water displacement method as in this study, the occupational leg swelling was 20 mL when using progressive elastic compression stockings (PECS) and 40 mL when using graduated compression elastic stockings (GECS).^7^ Although performing a direct comparison is difficult due to the different intervention methods and time periods, it was suggested that the insoles with a toe‐grip bar had similar effects to those of PECS and GECS.

In this study, the insoles with a toe‐grip bar increased the muscle activity of the tibialis anterior muscle and medial and lateral gastrocnemius muscles during the stance phase of walking, and the tibialis anterior muscle during the swing phase of walking. The insoles used in this study were composed of a toe‐grip bar made from synthetic fiber with high repulsive properties.^21^, ^22^, ^23^ In the terminal stance, the toes perceive the toe‐grip bar and the reflexive toe‐grip movement is increased. It has been reported that the muscle activity of the tibialis anterior and gastrocnemius muscles significantly increases and the tibialis anterior muscle acts as a fixator of the ankle joint while the gastrocnemius muscle acts as a cooperative muscle during toe gripping.^35^, ^36^ This suggests that the subconscious increase in toe‐grip movement during walking due to the toe‐grip bar promotes the muscle activity of the tibialis anterior and medial and lateral gastrocnemius muscles during the stance phase of walking. Moreover, the gastrocnemius muscle performs a crucial function to assist with the propulsive force when walking, achieved by the contraction of the muscles at the late stance phase in the gait cycle.^37^ Meanwhile, it has been reported that antagonist conditioning contraction (termed the “reversal of antagonists”) enhances subsequent agonist contractile activities.^38^, ^39^ Hence, these results suggest that the reversal of the muscle activity of the gastrocnemius muscle in the late stance phase, promoted by insoles with a toe‐grip bar, increases the muscle activity of the tibialis anterior during the swing phase.

There are some limitations to this study. First, participants who had no occupational leg symptoms were recruited in this study and walked approximately 4,000 steps. The effects of the insole on participants with occupational leg symptoms are unclear. Future studies need to verify the effect of wearing insoles with a toe‐grip bar in participants with occupational leg symptoms. Second, the change in walking style after wearing insoles with a toe‐grip bar, measured with temporo‐spatial gait parameters, has not been evaluated. Therefore, it cannot be denied that the unnatural walking style caused by wearing the novel insoles may increase the lower leg muscle activity. Future studies should examine the changes in gait parameters caused by insoles with a toe‐grip bar.

## 5 CONCLUSIONS

The findings of this study suggest that insoles with a toe‐grip bar may contribute to the attenuation of occupational leg swelling and increase lower limb muscle activity. Moreover, they can be widely applied as a simple tool to reduce leg swelling by simply walking.

## DISCLOSURE

*Approval of the research protocol*: The study was conducted according to the principles of the Declaration of Helsinki and was approved by the Institutional Ethics Committee of Kyoto Tachibana University. *Informed consent*: All participants provided their written informed consent, and they were allowed to withdraw from the study at any time. *Registry and the registration no. of the study/trial*: UMIN000041663. *Animal studies*: N/A. *Conflict of interest*: The authors declare no conflict of interest for this article.

## AUTHOR CONTRIBUTION

Conceptualization, S.M; methodology, SM; validation, HN, SM, YK, and TA; formal analysis, HN, YK, and TA; investigation, HN, SM, YK, and TA; resources, DM and MK; data curation, HN, YK, and TA; writing—original draft preparation, HN; writing—review and editing, HN and SM; visualization, HN; supervision, SM; project administration, SM All authors have read and agreed to the published version of the manuscript.

## Data Availability

The data that support the findings of this study are available from the corresponding author, upon reasonable request.
